# A guide to evaluating linkage quality for the analysis of linked data

**DOI:** 10.1093/ije/dyx177

**Published:** 2017-09-07

**Authors:** Katie L Harron, James C Doidge, Hannah E Knight, Ruth E Gilbert, Harvey Goldstein, David A Cromwell, Jan H van der Meulen

**Affiliations:** 1 Department of Health Services Research and Policy, London School of Hygiene & Tropical Medicine, London, UK; 2 Administrative Data Research Centre for England, UCL Great Ormond Street Institute of Child Health, UCL, London, UK; 3 Centre for Population Health Research, University of South Australia, Adelaide, Australia; 4 Lindsay Stewart Centre for Audit and Clinical Informatics, Royal College of Obstetricians and Gynaecologists, London, UK; 5 Graduate School of Education, University of Bristol, Bristol, UK

**Keywords:** Record linkage, linkage error, bias, hospital records, data accuracy, sensitivity and specificity, selection bias, data linkage, administrative data

## Abstract

Linked datasets are an important resource for epidemiological and clinical studies, but linkage error can lead to biased results. For data security reasons, linkage of personal identifiers is often performed by a third party, making it difficult for researchers to assess the quality of the linked dataset in the context of specific research questions. This is compounded by a lack of guidance on how to determine the potential impact of linkage error. We describe how linkage quality can be evaluated and provide widely applicable guidance for both data providers and researchers. Using an illustrative example of a linked dataset of maternal and baby hospital records, we demonstrate three approaches for evaluating linkage quality: applying the linkage algorithm to a subset of gold standard data to quantify linkage error; comparing characteristics of linked and unlinked data to identify potential sources of bias; and evaluating the sensitivity of results to changes in the linkage procedure. These approaches can inform our understanding of the potential impact of linkage error and provide an opportunity to select the most appropriate linkage procedure for a specific analysis. Evaluating linkage quality in this way will improve the quality and transparency of epidemiological and clinical research using linked data.

**Key Messages**

- Errors in data linkage are a potential source of bias in results of studies using linked data, yet researchers using linked data often find it difficult to assess the extent of such bias, due to the separation of linkage and analysis processes.
- We describe three methods for evaluating data linkage quality and identifying potential sources of bias: applying the linkage algorithm to a subset of gold standard data to quantify linkage error; comparing characteristics of linked and unlinked data to identify potential sources of bias; and evaluating the sensitivity of results to changes in the linkage procedure.
- These methods are relevant, however and by whoever the linkage is conducted, and can provide a better understanding of the pattern of any bias, the extent to which linkage error may affect our results, and determinants of the amount of bias that is likely to be introduced.
- When linkage error is identified as a possible source of bias, methods to adjust for these biases should be used, which can help provide more robust results.

## Introduction

Epidemiological and clinical research is increasingly based on datasets created by linking data from different sources such as administrative hospital datasets, clinical databases and national death registers.^1^^,^^2^ Due to the nature of many of these data sources, which are typically collected for financial or clinical management, a unique person identifier (e.g. National Health Service number in the UK) may not be available for linkage. Therefore, linkage is often based on a series of identifiers that are not unique, are prone to errors or missing values, or are dynamic (i.e. may change over time, such as postcode or name). Errors in the linkage process that arise from imperfect identifiers are increasingly being recognized as a potential source of bias in results from studies using linked data.^3^^,^^4^ However, assessing the extent to which linkage error affects results can be difficult for users of linked data who do not have access to the identifiable data used for linkage, which is often the case if the data linkage is performed by a third party. Separation of linkage and analysis processes in this way is recommended to preserve data security and personal confidentiality.^5^

Studies that evaluate linkage quality are therefore often restricted to estimates of the match rate (the proportion of records that were linked), sensitivity (the proportion of true links that were detected), or positive predictive value (the proportion of detected links that were true), which can be obtained, for example, by comparing a linked dataset with a ‘gold standard’ or reference dataset where true match status is known.^6^ However, these metrics are limited in their ability to tell us the degree to which linkage error might produce bias in outcomes of interest. In some instances, we can assume that effect sizes will be underestimated but, in most scenarios, it is not straightforward to predict the direction of bias that may result from linkage error. There are several factors that determine how linkage error affects an estimate, but one key factor is the distribution of errors with respect to variables of interest, and this is usually unknown. In complex analyses incorporating multiple variables, different variables can be affected by linkage error in different ways.

There is a lack of guidance on how to explore the extent to which error impacts upon analysis, and this area has been identified as a priority for research.^7^^,^^8^ This Education Corner article describes three simple approaches for evaluating quality of linkage, using an illustrative example of a linked dataset of maternal and baby hospital records. We aim to provide guidance that is applicable to both data providers and researchers, and to encourage the application of these methods among researchers using linked data.^9^

## Why is linkage error important?

Linkage error can occur in two ways: false matches and missed matches. False matches occur when records belonging to different individuals are erroneously linked together. False matches typically (but not always) add noise to estimates, diluting the association between variables captured in different datasets and biasing effect estimates towards zero.^3^ Missed matches occur when records belonging to the same individual are not linked. When unlinked records are excluded from analyses, one consequence is reduced sample size and statistical power. If linkage is ‘informative’ (e.g. linkage to a disease register indicating the presence of a particular condition), a consequence of missed matches can be under-ascertainment of exposures or outcomes.^10^^,^^11^

An important further issue is that linkage errors do not always occur randomly, meaning that particular subgroups of individuals are often over- or under-represented amongst records affected by linkage error. Systematic reviews of studies comparing the characteristics of linked and unlinked records have identified that more vulnerable or hard to reach populations are often missed, with the probability of a missed match being associated with a range of characteristics including gender, age, ethnicity, deprivation and health status.^12^^,^^13^ Consequently, the linked data may not be representative of the population of interest, which can reduce the study’s external validity, or may not capture subgroups that are of particular interest. As these demographic variables are often associated with exposures or outcomes of interest, differential rates of linkage error may also introduce bias. For example, unlinked mortality records in one particular ethnic group could lead to a distorted comparison of mortality rates by ethnicity.^14–16^

If unlinked records are to be excluded from analysis, selection bias (or collider bias) can occur if selection into the linked dataset is related to both an exposure and an outcome of interest.^17^^,^^18^ For example, suppose it is more difficult to link records for low birthweight babies and also more difficult to link records from mothers who smoke. In this case, records for low birthweight babies that are successfully linked are more likely to be from mothers who do not smoke (since, in this example, records from mothers who smoke are more difficult to link). Conditioning on linked records could therefore induce a protective relationship between maternal smoking and low birthweight, analogous to the birthweight paradox described in epidemiological literature.^19^

## Evaluating the impact of data linkage error

The following sections describe three approaches to evaluating linkage quality (see Box 1 for a summary). The use of these methods can help researchers using linked data to understand the potential impact of linkage error on results, and comprise:

- using a gold standard dataset to quantify false matches and missed matches;
- comparing characteristics of linked and unlinked data to identify potential sources of bias;
- using sensitivity analyses to evaluate how sensitive results are to changes in linkage procedure.

We use an illustrative example of linkage between hospital records for mothers and babies to demonstrate how these approaches can be implemented by researchers using linked data.

**Table:** Box 1. Summary of approaches to evaluating linkage quality

	Using a gold standard dataset to quantify false matches and missed matches	Comparing characteristics of linked and unlinked data to identify potential sources of bias	Sensitivity analyses to evaluate how sensitive results are to changes in linkage procedure
Purpose	To quantify errors (missed matches and false matches)	To identify subgroups of records that are more prone to linkage error and are potential sources of bias	Assesses the extent to which results of interest may vary depending on different levels of error, and the direction of likely bias
Strengths	Easily interpretable; allows linkage error to be fully measured	Straightforward to implement and easily interpretable	Straightforward to implement
Limitations	Representative gold standard data are rarely available	Cannot be applied if systematic differences are expected between linked unlinked records (e.g. if linking to death register)	Results may be difficult to interpret as false matches and missed matches may impact on results in opposing or compounding ways
Technical requirements	A representative group of records for which true match status is known; data linker capacity to perform evaluation (researchers rarely have access to gold standard data)	A linkage design where all records in at least one file are expected to link; provision of record-level or aggregate characteristics of unlinked records to researchers	Provision of information on the strength of the match (e.g. deterministic rule or probabilistic match weight)

### Illustrative example: linking hospital records for mothers and babies

The two most popular approaches to linkage have previously been described in an Education Corner article: deterministic (or rule-based) methods and probabilistic (or score-based) methods.^20^ Alternative methods also exist.^21^^,^^22^ In a previous study, we used a combination of these techniques to create a mother-baby cohort of records from Hospital Episode Statistics (HES), an administrative data resource that holds detailed information of all admissions to National Health Service (NHS) hospitals in England.^23^ The methods are described in full elsewhere, but comprised deterministic and probabilistic linkage of de-identified information in data items contained in both the mother’s delivery record and the baby’s birth record.^24^ Using a deterministic algorithm based on exact matching of hospital, maternal age, gestational age, baby’s sex, birth order and GP practice code, 42% of baby records were linked to a maternal record. The match rate increased to 98% through the use of probabilistic linkage and additional variables (e.g. admission dates and mode of delivery). However, the extent to which these missed matches (or any false matches) affect analyses has not yet been explored.

### Evaluating linkage error using ‘gold standard’ data

If data are available where the true match status of each pair of records is known, these ‘gold standard’ data can be used to test linkage algorithms and estimate rates of linkage error. There are various ways in which gold standard datasets can be derived, for example from an additional data source with complete identifiers, from a subsample of records that have been manually reviewed or otherwise determined to be matches (or non-matches), or from a representative synthetic dataset (e.g. generated through simulating data).^11^^,^^25^^,^^26^ Gold standard datasets allow us to identify: where errors have occurred in our linkage; where we have failed to link records that should have been linked (missed matches); or where we have linked together records belonging to different entities (false matches). Since gold standard data should be linked in the same way as the study data (for comparison), involvement of data linkers is required. Unlike the second two approaches, gold standard comparisons cannot be readily implemented by researchers who do not have access to the identifying data (e.g. in a ‘trusted third party’ model).

### Creating the gold standard dataset

**Figure 1:**
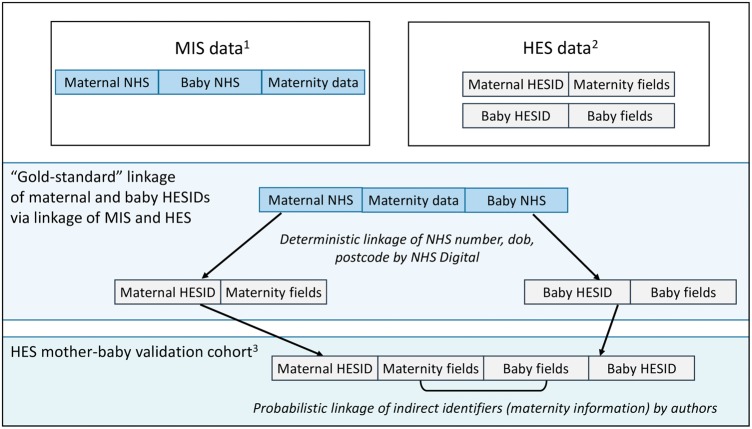
Creation of a gold standard dataset for evaluating linkage quality in the HES mother-baby cohort. ^1^83 195 records of births/deliveries from 15 English hospitals, April 2012–March 2013; ^2^672 955 records of births/deliveries from all NHS hospitals in England, April 2012–March 2013; ^3^72 817 records in the HES mother-baby validation cohort (gold standard).

In order to create a gold standard for assessing the quality of linkage within the HES mother-baby cohort, we needed a dataset where the true match status of HES maternal and baby records was known. This was possible due to another study, which collected electronic records from maternity information systems (MIS) within 15 English obstetric units for births between April 2012 and March 2013.^27^ The MIS data captured NHS numbers for both mothers and babies together on the same record (this is not the case for HES). The MIS records were linked by NHS Digital to corresponding maternal and baby records in HES, using a deterministic approach based on NHS number, date of birth, sex and postcode. After excluding a number of uncertain links (see Supplementary Figure 1, available as Supplementary data at *IJE* online), the MIS-HES links provided a gold standard dataset that could be used to validate the same subset of births in the linked HES mother-baby cohort (Figure 1).

### Evaluating linkage using the gold standard dataset

We compared linked records in the gold standard dataset (linked using direct patient identifiers including NHS number) with those in our HES mother-baby cohort (linked using indirect identifiers captured for birth and delivery records). To enable this comparison, we applied our original linkage algorithm to the same set of HES records captured in the MIS data. This allowed us to quantify the number of false matches or missed matches that our linkage algorithm produced, and to derive standard measures of linkage quality (sensitivity/recall, false match rate and positive predictive value/precision).^28^ These metrics are thought to be more useful than specificity (due to the imbalance between the number of non-matches versus matches in a linkage) or the F-measure (due to issues when comparing linked datasets of different sizes).

The gold standard dataset comprised records for 72 817 babies (Supplementary Figure 1). Of these, 72 520 (99.6%) were linked using the original linkage algorithm and 297 (0.4%) were unlinked. Of the 72 520 linked records, 71 884 were true matches, giving a positive predictive value of 99.1%, a false match rate of 0.9% (636 false matches/72 520 linked records) and a sensitivity of 98.7% (71 884/72 817 true matches).

The low error rates observed in this evaluation demonstrate that the original linkage algorithm was highly accurate. We might therefore assume that the impact of linkage error will be negligible, since, for example, excluding such a small proportion of the target population is unlikely to affect the generalizability of results or to dramatically reduce precision. However, further evaluation is needed to assess whether selection bias could be present, e.g. if records from a particular subgroup were more likely to be missed, or whether the 0.9% of false matches could have introduced enough noise to bias results.

### Comparing characteristics of linked and unlinked data

In order to identify particular subgroups of records that are differentially missed during linkage, we can compare the characteristics of linked and unlinked records. This method of quality appraisal can only be implemented when all of the records in at least one of the files (or within the target sample within one of the files) are expected to link; it would not be useful, for example, when linking to a register of deaths to determine mortality, as there would be expected systematic differences between those who link and those who do not. This approach can be implemented if researchers have access to record-level or aggregate information on the characteristics of unlinked records.

Since data linkage studies are often characterized by large sample sizes, standardized differences can be more informative than *P*-values for comparing unlinked and linked records. Standardized differences are calculated as the mean difference divided by the standard deviation, and can be easily calculated in statistical software packages (e.g. using the ‘stddiff’ command in Stata).^29^^,^^30^ Standardized differences of 0.2, 0.5 and 0.8 represent small, moderate and large standardized differences, respectively.^29^^,^^31^ This helps us to identify variables that may have been more affected by linkage error and are therefore potential sources of bias.^32^

### Evaluating linkage using comparisons of linked and unlinked records

Compared with true matches in the gold standard, the 297 records that failed to link (missed matches) and 636 baby records that linked to the wrong mother (false matches) were more likely to be: multiple births; or babies with lower gestational age, lower birthweight or more neonatal medical conditions; or babies born by caesarean section; or those of non-White ethnic background (Table 1). Records with linkage errors were also more likely to be from babies born to nulliparous mothers or mothers without pregnancy risk factors. Linkage errors were strongly driven by data quality, since records with one or more missing values were less likely to link.

**Table 1:** Characteristics of records in the HES mother-baby cohort according to linkage status derived from gold standard data

		True matches (*N* = 71884)		False matches (*N* = 636)		St. diff.	Missed matches (*N* = 297)		St. diff.
		*N*	%	*N*	%		*N*	%	
Stillbirth		325	0.5	6	0.9	0.1	9	3.0	0.2
Survival to postnatal discharge		71384	99.1	627	98.1	0.1	286	96.3	0.2
Delivery risk factor^a^		6738	9.4	105	16.4	0.2	49	16.5	0.2
Female infant		34967	48.7	321	50.2	0.0	140	47.1	0.0
Multiple birth		1961	2.7	126	19.7	0.6	31	10.4	0.3
Caesarean section		18034	25.1	63	9.9	0.4	10	3.4	0.7
Pregnancy risk factor^b^		7388	10.3	16	2.5	0.3	1	0.3	0.5
Neonatal medical condition^c^		6281	8.7	91	14.2	0.2	90	30.3	0.6
Neonatal ICU		8461	11.8	32	5.0	0.2	33	11.1	0.0
Parity: nulliparous		27125	37.7	335	52.4	0.3	192	64.5	0.6
Gestational age group	Full term (39+ wks)	45611	72.3	102	44.4	0.7	27	44.3	0.7
	Early term (37–38 wks)	12721	20.2	66	28.7		17	27.9	
	Late preterm (34–36 wks)	3280	5.2	39	17.0		6	9.8	
	Moderate/very preterm (< 34 wks)	1494	2.4	23	10.0		11	18.0	
	Missing*	8775	12.2	409	64.0		236	79.5	
Birthweight (g)	< 1500	909	1.4	14	6.1	0.7	7	10.9	0.7
	1500–< 2500	1798	6.0	45	19.7		12	18.8	
	2500–< 4000	51718	82.0	160	69.9		42	65.6	
	4000+	6687	10.6	10	4.4		3	4.7	
	Missing*	8769	12.2	410	64.2		233	78.5	
Size for gestation	Small (< 10th percentile)	5274	8.4	25	11.1	0.2	5	8.3	0.1
	Normal	54367	81.6	187	93.1		51	85.0	
	Large (> 10th percentile)	6344	10.1	13	5.8		4	6.7	
	Missing*	8896	12.4	414	64.8		237	79.8	
Ethnicity	White	48896	68.0	408	63.9	0.3	165	55.6	0.4
	Mixed	3410	4.7	24	3.8		14	4.7	
	Asian	7367	10.3	49	7.7		20	6.7	
	Black	4866	6.8	32	5.0		25	8.4	
	Other	4508	6.3	77	12.1		38	12.8	
	Unknown	2834	3.9	49	7.7		35	11.8	
Newborn length of stay (days)	< 2	38329	53.3	315	49.3	0.2	131	44.1	0.7
	2–6	28946	40.3	244	38.2		74	24.9	
	7+	4599	6.4	80	12.5		92	31.0	
Maternal age (years)	< 20	2859	4.0	21	3.3	0.1	13	4.4	0.2
	20–24	11752	16.4	88	13.8		42	14.1	
	25–29	19226	26.8	155	24.3		55	18.5	
	30–34	22377	31.1	220	34.4		101	34.0	
	35–39	12433	17.3	125	19.6		64	21.6	
	40+	3234	4.5	30	4.7		22	7.4	
Income/deprivation quintile^d^	Most deprived	27042	37.7	206	32.3	0.1	97	32.9	0.2
	2	16394	22.9	170	26.7		86	29.2	
	3	13104	18.3	129	20.3		58	19.7	
	4	9040	12.6	77	12.1		37	12.5	
	Most affluent	6146	8.6	55	8.6		17	5.8	
	Missing*	155	0.2	2	0.3		2	0.7	

0.2, 0.5, and 0.8 can be considered as small, medium and large effect sizes respectively.

St. diff, standardized differences; ICU, intensive care unit; wks, weeks.

^a^Hypoxia, amniotic fluid embolism, placental-transfusion syndrome, umbilical cord prolapse, chorioamnionitis, fetal haemorrhage, birth trauma, complications of delivery, umbilical cord problem.

^b^Eclampsia, gestational hypertension, diabetes, placental abruption or infarction.

^c^Congenital anomaly, perinatal infection, neonatal abstinence syndrome, respiratory distress syndrome.

^d^Quintiles of deprivation were derived from the Index of Multiple Deprivation (IMD) score based on patient postcode in HES.

*Percentage of records with missing data (excluded from other category percentages).

The results in this example indicate that although linkage error rates were low, there was still some potential for bias, as particular subgroups of records were more often affected than others. Whether these differences were large enough to introduce bias into results depends on the relationship between these variables and the parameters of interest. It is therefore helpful to explore how results of interest might change according to different levels of error.

### Sensitivity analyses

In order to assess the sensitivity of results to different linkage procedures, we can perform sensitivity analyses, aiming to assess the extent to which results vary and the direction of likely bias. This can involve changing the linkage algorithm or varying the match weight threshold for probabilistic linkage, and re-running analyses to evaluate any impact on results.^3^^,^^14^ The aim of this approach is to determine whether decisions about the design of the linkage procedure could have had a substantial impact on inferences drawn from the linked data. These types of sensitivity analysis can be implemented by researchers without access to identifying data, if they are provided with match weights (in probabilistic linkage) or decision steps (in deterministic linkage). As these are not sensitive data, data providers and linkers are usually able to share these with researchers.^9^

### Evaluating linkage using sensitivity analyses

We conducted a sensitivity analysis to evaluate the mother-baby linkage by changing the threshold used in our linkage algorithm, and comparing results across different sets of linked records. We compared linkage results from our original probabilistic algorithm using a threshold weight of 20 for classifying records as links, with results from an algorithm that minimized false links by using a considerably higher threshold of 45. These thresholds were selected based on examination of the observed distribution of weights in our analysis; this distribution can differ substantially depending on the number and quality of matching variables, so thresholds are generally selected in the context of a specific linkage or analysis. The aim of this type of sensitivity analysis is to select thresholds that are likely to reflect plausible limits for the trade-off between false matches and missed matches. We also compared results with those from the initial deterministic linkage only, i.e. where records agreed exactly on hospital, maternal age, gestational age, baby’s sex, birth order and GP practice code.

As expected, increasing the match weight threshold in probabilistic linkage, or using deterministic linkage only, produced linkages that introduced fewer false matches but more missed matches (Table 2). This is because stricter linkage criteria make it less likely that records belonging to different entities will link by chance, but more likely that records with missing or incorrect linking variables will remain unlinked.

**Table 2:** Linkage success for a range of linkage criteria

	Original probabilistic linkage (threshold weight = 20)	High-threshold probabilistic linkage (threshold weight = 45)	Deterministic linkage only
Linked records	72520/72817	65020/72817	35324/72817
	99.6%	89.3%	48.5%
Missed match rate	297/72817	7797/72817	37493/72817
	0.4%	10.7%	51.5%
False match rate	636/72520	212/65020	22/35324
	0.9%	0.3%	0.1%
Positive predictive value	71884/72520	64808/65020	35302/35324
	99.1%	99.7%	99.9%

We expected that impact of linkage errors in each of these linkage scenarios would depend on the research question, and therefore assessed four different outcomes: proportion of stillbirths; the proportion of preterm births (< 37 weeks of gestation); the association between neonatal survival to discharge and delivery risk factors; and the association between delivery risk factors and ethnic group. Odds ratios were estimated from logistic regression models, adjusting for a number of maternal and neonatal risk factors (listed in Table 1, based on ICD-10 diagnosis codes listed in Supplementary Table 1, available as Supplementary data at *IJE* online). Analysis was performed in Stata 14.^33^

#### Proportions of stillbirths and preterm births

We expected that the generalizability (i.e. external validity) of the data would be affected by missed matches. In particular, we expected that records of preterm births or stillbirths would be less likely to link than those of later gestations or live births, and that the ascertainment of these outcomes would therefore be lower in datasets more affected by missed matches.

By comparing results across different linkage algorithms (Table 3), we observed that for preterm birth (7.65% of records in gold standard), ascertainment was similar for the original linkage algorithm (7.64%), but ascertainment was a little lower when using a higher match weight threshold of 45 (7.31%) or deterministic linkage only (7.43%). All confidence intervals for proportions of stillbirths and preterm births estimated in the linked datasets overlapped with those in the gold standard, indicating that in this example, linkage error is unlikely to have resulted in substantial bias for these outcomes.

**Table 3:** Comparison of outcome measures for a range of linkage criteria

	Gold standard	Original probabilistic linkage	High-threshold probabilistic linkage	Deterministic linkage only
% Preterm births (95% CI)	7.65 (7.45–7.86)	7.64 (7.43–7.85)	7.31 (7.11–7.53)	7.43 (7.16–7.71)
% Stillbirths (95% CI)	0.47 (0.42–0.52)	0.46 (0.41–0.51)	0.44 (0.39–0.49)	0.45 (0.40–0.50)
Odds ratio (95% CI) for neonatal survival to discharge: mothers with delivery risk factors vs those without	0.40 (0.17–0.95)	0.42 (0.18–0.98)	0.35 (0.15–0.79)	0.52 (0.22–1.25)
	*P* = 0.039	*P* = 0.044	*P* = 0.011	*P* = 0.143
Odds ratio (95% CI) for delivery risk factors: Black ethnicity vs White ethnicity	0.98 (0.88–1.09)	0.97 (0.87–1.08)	0.89 (0.79–1.01)	0.80 (0.66–0.96)
	*P* = 0.700	*P* = 0.593	*P* = 0.067	*P* = 0.017

#### Association between neonatal survival to discharge and recording of delivery risk factors

We expected that statistical power would be affected either through missed matches (due to a reduction in the size of the study population) or a lack of precision introduced by false matches (leading to increased noise in the association between variables). Given the large sample size, we assumed that power implications would be most important for identifying associations with rare outcomes (e.g. mortality).

**Figure 2:**
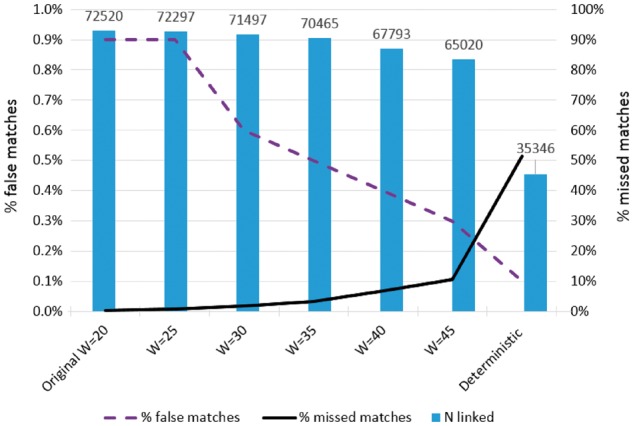
Number of linked records and percentage of missed matches and false matches for a range of linkage criteria. W = threshold used to classify links in probabilistic linkage.

As expected, we found that as the number of linked records decreased due to more missed matches at higher thresholds (Figure 2), there was a reduction in the precision of estimates of association between delivery risk factors and survival (Table 3). Although an association between delivery risk factors and survival was observed in the gold standard data [adjusted odds ratio = 0.40; 95% confidence interval (CI) 0.17–0.95], this association was no longer observed in the deterministic linkage where less than half of records were linked (adjusted odds ratio = 0.52; 95% CI 0.22–1.25). These results demonstrate that there can be power implications when large numbers of unlinked records are excluded from analysis: a problem analogous to that of complete case analysis in the presence of missing data.

#### Association between delivery risk factors and ethnicity

We expected that selection bias could be introduced if selection into the linked dataset were associated with both the outcome and exposure. For example, earlier comparisons between linked and unlinked data had indicated that that records for mothers with delivery risk factors were less likely to link, and also that mothers in the Black ethnic group were less likely to link. If this was the case, mothers with delivery risk factors who were successfully linked would be more likely to be from other ethnic groups (since, in this example, those from the Black ethnic group were harder to link). Conditioning on linked records could therefore induce a spurious protective relationship between Black ethnicity and delivery risk factors.

In the gold standard data, we observed that 6.5% of mothers with delivery risk factors were from the Black ethnic group, whereas in the deterministic linkage only 4.7% of mothers with delivery risk factors were from the Black ethnic group. There was no true association between ethnicity and delivery risk factors. However, within the deterministically linked data there appeared to be a protective effect (odds ratio = 0.80; 95% CI 0.66–0.96, Table 3). These results indicate that in this example, some inferences from linked data could be incorrect due to selection bias in the presence of missed matches.

## Discussion

We describe three approaches for evaluating linkage quality, and demonstrate how these methods can be used determine the extent to which linkage error may introduce bias for a specific research question. Our illustrative example showed that even with high linkage rates, particular subgroups of individuals are disproportionately affected by linkage error, as has been observed in previous literature.^13^ However, we demonstrate that sensitivity analyses can help us to understand the direction of any bias, and to assess whether linkage errors may influence inferences from the linked dataset.

### Access to information required to evaluate linkage quality

In many jurisdictions, linkage is carried out by an independent body and information about the linkage processes is not readily available to researchers. Comprehensive guidelines on information that should be shared between data providers, linkers and researchers are available elsewhere.^9^ All of the approaches described in this article require some level of collaboration between data linkers and the researchers aiming to evaluate linkage quality, but only approach (i) (gold standard data) requires direct involvement of the linkers; approaches (ii) and (iii) can be implemented by researchers provided that certain non-sensitive information is shared.

Our example of HES mother-baby linkage was supported by the availability of a subset of gold standard data, which is one of the most useful tools for quantifying linkage error. Consideration should always be given to the representativeness of the gold standard dataset. In our example, the proportion of unlinked records in the gold standard data was lower than in the HES mother-baby cohort overall, indicating that data quality in the hospitals contributing MIS data may have been slightly better than average. The same applies to gold standard datasets created using subsets of records that have complete data either on a single unique identifier or on a sufficient set of partially identifying variables; records with high quality data may differ systematically from those of poorer quality data.

In practice, gold standard datasets are rarely available. Even those that are generated (e.g. through manually reviewing a sample of linked and unlinked records, or by creating a synthetic dataset with the same characteristics as the original data) are often only available to the data linkers and not to researchers.^34^ This means that data linkers would need to evaluate linkage quality using a gold standard (as researchers generally would not have access to gold standard data).

However, when gold standard datasets are not available, researchers can consider alternative approaches: comparisons of characteristics of linked and unlinked data, and sensitivity analyses. These methods can be easily implemented but require data linkers to provide information on the characteristics of unlinked records and/or on the quality of each potential link.^9^ Providing record-level or aggregate characteristics of unlinked records allows researchers to compare linked and unlinked records, to identify any potential sources of bias where particular subgroups of records were missed from the linkage.

Sensitivity analyses can be performed if measures of linkage certainty (e.g. match weights in probabilistic matching or matching ranks/criteria in hierarchical deterministic matching) are provided by data linkers alongside a linked dataset.^9^ This makes any uncertainty or subjectivity in the linkage process more transparent and allows researchers to run analysis on different sets of linked records in turn. It should be noted that caution is needed when interpreting results of this type of sensitivity analysis, as in most cases, none of the linkage algorithms compared will be 100% accurate. The trade-off between false matches and missed matches will vary depending on the linkage algorithm, and these errors may impact on results in opposing or compounding ways. In our example, inferences about the associations between delivery risk factors and survival to discharge, and between ethnicity and delivery risk factors, differed between the methods. Exploring differences in results over a range of linkage algorithms in this way can help researchers consider the pattern of any bias, and to identify scenarios which are particularly likely to produce substantial bias.

### Further methods for evaluating linkage quality

In our example, we expected all babies to link with a mother, which made comparisons between linked and unlinked records easily interpretable and allowed us to directly estimate the proportion of missed matches. However, careful consideration needs to be given to appropriate reference populations when all records are not expected to link. For example, we would not expect all hospital records to link with a mortality record and vice versa; rather, a successful link indicates that an individual has died (‘informative linkage’). If that is the case, comparing the characteristics of the individuals whose records were and were not linked would also be affected by differences in the groups for whom no linked records were available (i.e. the difference between those who died and those who survived). In such situations, external reference data (e.g. age-specific mortality rates) can allow us to assess how linkage rates might differ for different subgroups.^10^

Further methods not covered in this article can also be used to evaluate linkage quality in the absence of a gold standard. For example, estimates of false match rates can be derived by applying linkage algorithms to records known to have no match (e.g. attempting to link with mortality records for individuals known to be alive, or attempting to link male patient records with maternity records).^3^ Alternatively, inconsistency checks, such as checks for admissions following a patient’s death, linkage between a male patient and a caesarean section, or linkage of one mortality record to two different individuals, can be performed post-linkage in de-identified data to identify false matches.^35^^,^^36^ Linkage error rates estimated in this way should be interpreted with caution, as not all errors may have been identified and distinguishing between linkage errors and data coding errors can be difficult.^35^ Nevertheless, these methods can reveal useful information about the relative distribution of errors across subgroups or with respect to variables of interest (i.e. whereas the absolute error rate may remain unknown, higher rates of inconsistencies may be observed with respect to some variable of interest, implicating likely bias).^35^

### Handling bias in the analysis of linked data

Evaluation of linkage quality can guide decisions about appropriate study design. For example, if linkage is used to identify individuals with a particular condition or disease (informative linkage), high levels of missed matches will lead to under-ascertainment, meaning that cohort study designs may be unsuitable (particularly for deriving estimates of prevalence or incidence). Where linkage rates are too low, researchers may conclude that linked data are not fit for these purposes. On the other hand, a case-control study may still be valid, whereby a high threshold is used to identify cases and a low threshold is used to identify controls (assuming no other biases are present).^37^ In this scenario, records for which there is uncertainty about linkage would not be included in analysis.

An alternative, which still makes use of all available records, is to use multiple imputation to handle missing values due to unlinked or equivocal records.^38^ Furthermore, information from match weights can be incorporated into imputation procedures, making use of variable distributions in candidate links (known as ‘prior-informed imputation’).^39^^,^^40^ This method incorporates information from ‘auxiliary’ variables, such as individual characteristics associated with linkage quality (e.g. birthweight or ethnicity) to help correct for selection biases.

In situations in which we have information about how linkage error affects the distribution of outcomes and exposures in our data, it may be possible to use well-established techniques for quantitative bias analysis, to adjust for these errors.^41^^,^^42^ This is particularly relevant for simple analyses, but becomes more complex with complicated designs involving more than two data sources and/or a number of covariates. Developing appropriate methods to handle bias arising from linkage error is a priority for methodological research.^43^

Studies of linked data are often based on administrative data that have not been collected primarily for research. In addition to linkage error, researchers should also consider other issues specifically relevant to these types of data (e.g. missing data, coding changes, service changes etc.), and explore methods to handle any potential bias that is identified.^43^^,^^44^

### Summary

We describe three methods for evaluating linkage quality: applying the linkage algorithm to a subset of gold standard data to quantify linkage error; comparing characteristics of linked and unlinked data to identify potential sources of bias; and evaluating the sensitivity of results to changes in the linkage procedure. These methods are generalizable to many other linkage situations and can be used as a guide for evaluating the quality of linkage for population-based analyses of linked data. Researchers using linked data should collaborate with data providers to understand the data linkage process, including data extraction and cleaning, linkage methods and resulting data quality.^9^ Ultimately, this will improve transparency and enhance the value of linked data for epidemiological and clinical research.^44^

## Supplementary Data

Supplementary data are available at *IJE* online.

## Funding

This work was supported by the Wellcome Trust [grant number 103975/Z/14/Z]. JvM is supported by the NIHR CLAHRC North Thames. The work was also supported by the Economic and Social Research Council through the Administrative Data Research Centre for England (ES/L007617/1).

## Supplementary Material

Supplementary Data
