# Supplementary material for: A guide to evaluating linkage quality for the analysis of linked data
Source: Int J Epidemiol. 2017 Sep 7;46(5):1699–710. doi: 10.1093/ije/dyx177 (PMC5837697; doi:10.1093/ije/dyx177)
Supplement: Supplementary Data [file dyx177_ije-2017-03-0289-file006.docx]

# Supplementary material

**Supplementary Figure 1: Creating the gold-standard data from linkage between MIS and HES.** Linkage was performed by NHS Digital.

^1^ Records were not present in the HES maternity or birth cohorts if they had missing episode end date, were terminations, or if there were HESID assignment errors (multiple individuals sharing a single HESID or one individual with multiple HESIDs)

^2^ Probable false-matches were identified as HES records that were discordant with MIS on at least 3 of the following: gestational age (within 1 week), birthweight (within 100 grams), baby’s date of birth (within 2 days), mother’s age (within 1 year), sex of baby. Missing values were not treated as discordant.

**Supplementary Table 1: ICD 10 code lists for pregnancy, delivery and neonatal risk factors**

| **Group** | **Description** | **ICD10 codes** |
| --- | --- | --- |
| Pregnancy risk factor | Intrauterine fetal death | O364, P95 |
|  | Eclampsia | O14,O15 |
|  | Gestational hypertension | O13,O16 |
|  | Placental abruption or infarction | O45,O431, O438, O439 |
|  | Uterine rupture | O710, O711 |
|  | Diabetes in pregnancy | P700, O24, E10-E14 |
| Delivery risk factor | Birth trauma | P10-P15 |
|  | Complications of delivery | P03 |
|  | Hypoxia | P20-P21 |
|  | Amniotic fluid embolism | O881 |
|  | Chorioamnionitis | O411 P027-P029 |
|  | Umbilical cord problem | P020, P024-P026 |
|  | Fetal hemorrhage | P50, P51, P53, P54 |
|  | Maternal hemorrhage | O430 |
|  | Umbilical cord prolapse | O69 |
| Neonatal medical condition | Congenital anomalies | Q00-Q07, Q10.4, Q10.7, Q11-Q12, Q13.0-Q13.4, Q13.8, Q13.9, Q14-Q16, Q20-Q26, Q18.8, Q30-Q37, Q38.0, Q38.3, Q38.4, Q38.6-Q38.8, Q39, Q40.2, Q40.3, Q40.8, Q40.9, Q41, Q42, Q43.1, Q43.3-Q43.7, Q43.9, Q44, Q45, Q50.0, Q51, Q52.0-Q52.2, Q52.4, Q54.0-Q54.3, Q54.8, Q54.9, Q55.0, Q55.5, Q56, Q60.1, Q60.2, Q60.4-Q60.6, Q61, Q62.0-Q62.6, Q62.8, Q63.0-Q63.2, Q63.8, Q63.9, Q64, Q65.0-Q65.2, Q65.8, Q65.9, Q67.5, Q68.2, Q68.3-Q68.5, Q71-Q73, Q74, Q75.0, Q75.1, Q75.3-Q75.9, Q76.1-Q76.4, Q77, Q78, Q79.0, Q79.2-Q79.5, Q79.6, Q79.8, Q82.0-Q82.4, Q82.9, Q86.2, Q85, Q86.0, Q86.1, Q86.8, Q87.8, Q89.1, Q89.2, Q89.3, Q89.7-Q89.9, Q90-Q93, Q95.2, Q95.3, Q97, Q99 |
|  | Complex chronic conditions | B20-B23, D55, D561, D562, D570-D572, D58, D80-D84, D898, D899, E343, E70-E730, E74, E76-E79, E803-E807, E83-E85, E881, E882, E888, E889, F70, F72, F73, F842, G10-G12, G20, G23, G240-G242, G248, G250-G256, G318, G319, G40, G41, G71, G72, G80-G82, G901, G903, G904, G91, G940-G942, G95, G99, I42, I44, I45, I47-I49, I515, K44, K50-K51, K73-K74, K754, K758-K760, M41, N18, P27, P90 |
|  | Neonatal abstinence syndrome | P961 |
|  | Noxious influences | P04 |
|  | Perinatal infection | P35-P39 |
|  | Meningitis or encephalitis | G00-G09 |
|  | Necrotising enterocolitis* | P77 |
|  | Intraventricular hemorrhage* | P52, P912 |
|  | Retinopathy of prematurity* | H351 |
|  | Respiratory distress syndrome* | P22 |

*Only included in this group if infants were born <37 weeks gestation.
